# From scanners to cell phones: neural and real-world responses to social evaluation in adolescent girls

**DOI:** 10.1093/scan/nsab038

**Published:** 2021-03-26

**Authors:** Stefanie L Sequeira, Jennifer S Silk, Elizabeth A Edershile, Neil P Jones, Jamie L Hanson, Erika E Forbes, Cecile D Ladouceur

**Affiliations:** Department of Psychology, University of Pittsburgh, Pittsburgh, PA 15213, USA; Department of Psychology, University of Pittsburgh, Pittsburgh, PA 15213, USA; Department of Psychology, University of Pittsburgh, Pittsburgh, PA 15213, USA; Department of Psychiatry, University of Pittsburgh, Pittsburgh, PA 15213, USA; Department of Psychology, University of Pittsburgh, Pittsburgh, PA 15213, USA; Department of Psychiatry, University of Pittsburgh, Pittsburgh, PA 15213, USA; Department of Psychiatry, University of Pittsburgh, Pittsburgh, PA 15213, USA

**Keywords:** adolescence, peers, fMRI, ecological momentary assessment

## Abstract

While expanded use of neuroimaging seemed promising to elucidate typical and atypical elements of social sensitivity, in many ways progress in this space has stalled. This is in part due to a disconnection between neurobiological measurements and behavior outside of the laboratory. The present study uses a developmentally salient fMRI computer task and novel ecological momentary assessment protocol to examine whether early adolescent females (*n *= 76; ages 11–13) with greater neural reactivity to social rejection actually report greater emotional reactivity following negative interactions with peers in daily life. As hypothesized, associations were found between reactivity to perceived social threat in daily life and neural activity in threat-related brain regions, including the left amygdala and bilateral insula, to peer rejection relative to a control condition. Additionally, daily life reactivity to perceived social threat was associated with functional connectivity between the left amygdala and dorsomedial prefrontal cortex during rejection feedback. Unexpectedly, daily life social threat reactivity was also related to heightened amygdala and insula activation to peer acceptance relative to a control condition. These findings may inform key brain–behavior associations supporting sensitivity to social evaluation in adolescence.

Neuroimaging methods are often used to study brain function supporting various psychological processes, but most neuroimaging tasks bear little resemblance to the real world (e.g. displaying angry faces without context). Further, the links between neural activity and daily subjective experiences or behaviors are rarely examined directly (Hasson and Honey, 2012; Wilson *et al.*, 2014). Thus, the ecological validity and real-world relevance of neuroimaging work are often unclear and leaves us wondering: What does an individual’s neural activity actually tell us about how that individual is likely to function in everyday life?

This question is especially important when considering neural processing of social and emotional information, information that is complex, contextual and critical for navigating the real world. This question is also particularly relevant to pursue for early adolescent females, a population highly sensitive to social evaluation (Rudolph and Conley, 2005). Heightened sensitivity to social evaluation, resulting in part from increased time spent with peers and changes in brain function (Crone and Dahl, 2012; Somerville, 2013), may be developmentally normative and help adolescents navigate complex social environments. However, high sensitivity to social threat specifically could contribute to alarming increases in psychopathology, particularly social anxiety, occurring during adolescence for females (Silk *et al.*, 2012a). Linking neural correlates of sensitivity to social threat to real-world perceptions of social threat could ultimately inform biobehavioral targets for adolescent mental health intervention.

Linking fMRI to everyday social behavior, however, carries challenges. Traditional psychosocial questionnaire measures, frequently used in tandem with fMRI, may not accurately portray daily functioning (Trull and Ebner-Priemer, 2014). Combining fMRI and ecological momentary assessment (EMA) methods has helped address this gap and provided initial insight into brain–behavior associations supporting socioemotional processing (e.g. Eisenberger *et al.*, 2007; Masten *et al.*, 2012). EMA involves repeated sampling of an individual’s affect and behavior in naturalistic conditions, which can provide rich insight and reduce retrospective bias (Wilson *et al.*, 2014). fMRI is a neuroimaging tool that can be used to study the neural substrates of nonverbal affective processing and taps more objective, biological phenomena (Wilson *et al.*, 2014). However, the ecological validity of much fMRI research is unclear. Integrating EMA and fMRI allows one to bridge brain in the laboratory and behavior in more naturalistic settings, to potentially increase the generalizability and clinical relevance of both fMRI and EMA findings.

Given the highly controlled, artificial environment of the scanner, it is also necessary to have ecologically valid scanner-based tasks that map more closely onto how individuals actually process socioemotional information in everyday life. One common task used to study neural processing of social threat in adolescence is the Cyberball task (e.g. Masten *et al.*, 2009; Will *et al.*, 2016; Rudolph *et al.*, 2016), a task in which participants believe they are being excluded during a virtual ball-tossing game (Williams *et al.*, 2000). Cyberball provides a critical interactive social component but fails to capture the modern, ‘online’ adolescent social context. Development of the Chatroom task (Guyer *et al.*, 2008, 2009), Chatroom Interact task (Silk *et al.*, 2012b) and Virtual School (Jarcho *et al.*, 2013) provide significant improvements in ecological validity. For example, during the Chatroom Interact task, adolescents enter an interactive online chatroom with virtual ‘peers’ whom they believe to be other participants in the study. Given the importance of peers and the pervasiveness of online social rejection for today’s youth, the Chatroom Interact task is a simple yet developmentally salient task to be used with fMRI methods.

Early fMRI work using these tasks has identified normative increases in neural activation to social rejection from childhood to adolescence in a social–affective brain network (see Somerville, 2013 for review). This network, which includes the amygdala, anterior cingulate cortex (ACC) and anterior insula, may be important for detecting and interpreting social cues and modulating affective responses to these cues (Jarcho *et al.*, 2013). Notably, while the dorsal ACC has been noted as a core region of the ‘social pain’ network (Eisenberger, 2012), this region has not been identified in meta-analyses of social rejection (Cacioppo *et al.*, 2013; Vijayakumar *et al.*, 2017; Mwilambwe-Tshiloboa and Spreng, 2021). However, the subgenual ACC (sgACC) has been reliably linked to social rejection processing, particularly in youth (Rotge *et al.*, 2015; VijayaKumar *et al.*, 2017). Day-to-day implications of patterns of neural activity during ecologically valid tasks, such as Chatroom Interact, remain largely unknown. One possibility is that youth with greater neural reactivity to peer rejection find negative experiences with peers more salient and distressing in daily life. This interpretation may be supported by initial research showing associations between adolescents’ neural activity to social rejection and self-reported rejection sensitivity (Burklund *et al.*, 2007; Masten *et al.*, 2009).

This interpretation may also be supported by a handful of studies that have linked neurobiological measures of social threat reactivity to EMA measures of socioemotional processing in youth (e.g. Forbes *et al.*, 2009; Masten *et al.*, 2012; Silk *et al.*, 2012b; Price *et al.*, 2016; Oppenheimer *et al.*, 2020). For example, Silk *et al.* 2012b found that youths with higher physiological arousal (measured via pupillary response) to peer rejection on the Chatroom Interact task also reported lower feelings of social connectedness with peers in daily life. The relationship between neural sensitivity to rejection and socioemotional functioning is likely reciprocal, such that daily social and emotional experiences also influence brain function. In support of this, Masten *et al.* (2012) showed that greater involvement with friends during adolescence (measured via daily diary methods) was associated with less neural reactivity to social rejection on the Cyberball task in young adulthood. Additionally, adolescents with a history of childhood peer victimization show stronger activation in the social–affective brain network to peer rejection relative to non-victimized adolescents (Will *et al.*, 2016; Rudolph *et al.*, 2016).

Despite several recent studies examining the neural processing of social rejection, and promising initial work linking EMA to neurobiology in adolescence, no studies have yet linked concurrent measures of fMRI and EMA to examine how neural processing of social rejection relates to daily socioemotional functioning in adolescence. This approach has, however, been taken in adults. Eisenberger and colleagues (2007) showed that adults with greater activation in regions associated with processing social threat (i.e. ACC, amygdala and periaqueductal gray) during the Cyberball task reported greater disconnection and rejection during real-world social interactions. This was a critical finding linking brain and daily behavior. Given the complexities of human social interactions, however, the field requires a more nuanced examination of potential emotions and cognitions associated with negative social interactions in daily life. Further, it is unknown how these findings might replicate during adolescence, an important developmental period in which to study brain–behavior associations supporting sensitivity to social threat. Many have hypothesized that maturation in the social–affective brain network supports increased sensitivity to social evaluation during adolescence (e.g. Nelson *et al.*, 2005; Somerville, 2013); identifying real-world correlates of neural reactivity to social threat may provide evidence to support this hypothesis.

Addressing these limitations, the present study used novel, ecologically valid techniques to examine associations between fMRI findings and perceptions of daily social threat in early adolescent females oversampled for shy/fearful temperament. This sample was chosen to enrich variability in threat responding, as children and adolescents reporting subclinical symptoms of anxiety report more negative peer interactions and victimization (e.g. Hodges and Perry, 1999; Goldbaum *et al.*, 2003). Temperamentally shy and fearful girls are also at risk for developing future social anxiety disorder (Chronis-Tuscano *et al.*, 2009). Studying brain–behavior associations supporting sensitivity to social threat in temperamentally shy/fearful early adolescent females may provide insight into the etiology of social anxiety disorder in later adolescence.

In addition to capturing the modern social environment using the Chatroom Interact task, we assessed nuances of everyday peer interactions using a newly developed set of EMA items. These items capture negative self-oriented thoughts and feelings associated with perceptions of socially threatening peer experiences. For example, adolescents were asked whether they felt criticized, embarrassed, disliked and/or left out following negative interactions they had with peers throughout the day. Multilevel exploratory factor analyses were conducted to examine the structure of a perceived social threat scale using these items. Assuming adequate reliability, we created an average social threat score for each participant, with higher social threat scores indicating greater emotional reactivity to perceived social threat in daily life. We hypothesized that greater activation in a social–affective brain network (i.e. amygdala, sgACC and anterior insula) to peer rejection in the scanner would predict higher levels of reactivity to perceived social threat in daily life.

Associations between perceived social threat in daily life and functional connectivity between the amygdala and medial prefrontal cortex (mPFC) during rejection feedback were also examined. We focused on fronto-amygdala circuitry given the known role of the PFC in emotion regulation broadly (Banks *et al.*, 2007) and regulating the amygdala’s response to threats in the environment specifically (Ochsner *et al.*, 2002; Hariri *et al.*, 2003). More negative amygdala–mPFC connectivity during threat may represent a mature pattern of connectivity (Gee *et al.*, 2013); youths with stronger negative amygdala–mPFC connectivity may be more effectively recruiting prefrontal regions to exert top-down control over an initial, bottom-up amygdala response to threat (Quirk and Beer, 2006). We hypothesized that youth reporting greater emotional reactivity to perceived social threat in daily life would show reduced negative fronto-amygdala connectivity during rejection, potentially reflecting an inability to effectively regulate social threat in the laboratory and in the real world.

## Methods

### Participants

Seventy-six girls (*M*_age_ = 12.27 years, s.d. = 0.81 years) were included in analyses for the present study. Participants were recruited for a longitudinal study of girls’ brain development via advertisements and announcements in the community. Participants were oversampled for shy/fearful temperament to enrich variability in threat responsivity. A total of 522 families responded to recruitment efforts and completed a brief phone or web-based screen. Of these, 235 girls aged 11–13 met preliminary inclusion criteria and were scheduled for an initial clinical interview to determine eligibility, and 197 of these participants completed the initial visit (see online supplemental methods for more information).

Exclusion criteria included the presence of any past or current DSM-5 anxiety disorder (with the exception of specific phobia), major depressive disorder (MDD), attention-deficit/hyperactivity disorder (predominantly hyperactive–impulsive presentation or combined presentation), autism spectrum disorder, bipolar disorder or schizophrenia. Diagnostic status was determined at the initial visit using the Kiddie-Schedule for Affective Disorders and Schizophrenia-Present and Lifetime version (K-SADS-PL; Kaufman *et al.*, 1997), a semi-structured diagnostic interview administered to all participants by a trained clinician. All participants had an IQ > 70 as assessed using the verbal and matrix reasoning subtests of the Wechsler Abbreviated Scale of Intelligence (WASI; Wechsler, 2011). Additional exclusionary criteria include a lifetime presence of a neurological or serious medical condition, the presence of any MRI contraindications (e.g. dental braces, metal in the body and claustrophobia), uncorrected visual disturbance, presence of head injury or congenital neurological anomalies (based on parent report), acute suicidality, or taking medications that affect the central nervous system (e.g. selective serotonin reuptake inhibitors).

Of 197 participants who completed the initial visit, 129 were eligible for the study. The primary reason for exclusion was a current or lifetime history of an anxiety disorder or MDD. Of 129 eligible participants, 119 girls completed the fMRI scan for the current study; data from 25 participants were unusable due to excess movement (*n *= 19), falling asleep in the MRI scanner (*n *= 5) or incidental findings from the MRI scan that impeded analysis (*n *= 1). Of the 94 participants with usable fMRI data, EMA data were available for 90 participants; 14 of these 90 participants (15.6%) had unusable EMA data due to EMA drop-out (*n *= 3), low completion rate (<25% of EMA observations completed; *n *= 2), technical issue with the EMA phone (*n *= 1), or reporting less than three usable negative interactions with peers (*n *= 8). Excluded participants did not differ from included participants by age, total income, shy/fearful temperament (assessed using the Early Adolescent Temperament Questionnaire-Revised, EATQ-R; Ellis and Rothbart, 2001), anxiety symptoms (assessed using the Screen for Child Anxiety Related Emotional Disorders; Birmaher *et al.*, 1997), or depressive symptoms (assessed using the Mood and Feelings Questionnaire; Angold and Costello, 1987) (*P* values > 0.20). Included participants were predominately (71%) white. Median total family income in this sample was between $80 000 and $90 000. Key demographic characteristics are summarized in Table 1.

**Table 1.** Demographic characteristics of the total sample (*n* = 76)

	*n* (%)	Mean (s.d.)	Median	Range
Age		12.27 (0.81)	12.24	11.05–13.97
Total family income		7.17 (2.98)	8.00	0–10
Race/Ethnicity				
White	54 (71.1%)			
Black or African-American	13 (17.1%)			
Asian	2 (2.6%)			
Biracial	5 (6.6%)			
Other	2 (2.6%)			
Hispanic or Latino	3 (3.9%)			

*Note*: Total family income was reported by parents on a scale of 0–10 in increments of $10 000 (e.g. 0 = $0–10 000, 1 = $10 001–20 000…10 = $100 001+).

### Measures

#### Temperament.

Temperament was assessed using identical child and parent (parent report on child) versions of the EATQ-R (Ellis and Rothbart, 2001). In this sample, internal consistency for the EATQ-R shyness scale was moderate for adolescent self-report (Cronbach’s α = 0.74) and high for parent report (α = 0.87). Internal consistency for the EATQ-R fear scale was low for adolescent self-report (α = 0.46) and parent report (α = 0.66). The sample was stratified such that two-thirds of participants in this sample (*n *= 49) had scores higher than 0.75 s.d. above the mean on the parent- or child-rated shyness scale (2.99 for parent-report, 3.16 for child-report) or fear scale (3.12 for parent-report, 3.48 for child-report) All other participants scored below 0.75 s.d. above the mean on the fear and shyness scales of both child and parent reports.

#### Ecological Momentary Assessment (EMA).

Data on real-world social threat experiences were collected using cell phone EMA. Youth were given a pre-programmed android smartphone on which they entered responses to a series of questions about their daily experiences with peers using a secure smartphone app for Web Data Express developed by the Office of Academic Computing in the Department of Psychiatry of University of Pittsburgh.

Using these phones, participants were asked to answer questions about their most recent social interactions and their emotional responses to these interactions for 16 consecutive days. Adolescents were randomly sampled (i.e. received an electronic notification to respond) three times per day on weekdays (once in the morning between 7 AM and 8 AM and twice between 4 PM and 9:30 PM) and four times per day on the weekends between 10 AM and 9:30 PM, allowing for a maximum of 54 observations. This large number of samples allows for a more stable estimate of ‘typical functioning,’ even in the potential presence of several atypical days. Compliance in this sample was 81.3% (s.d. = 13.9%, range = 37.0–100%).

After receiving the electronic notification, adolescents were prompted through a series of questions about their recent emotions and interactions with friends. At the start of each observation, participants were asked to indicate how they were feeling ‘just before the phone beeped’ using a 0–100 sliding scale and various emotion words adapted from Silk *et al.* (2011). At each observation, ratings for sad, worried, stressed and mad were averaged to create a measure of momentary negative affect (NA). At each observation, ratings for happy, joyful, excited and interested were averaged to create a measure of momentary positive affect (PA). Reliability was moderate to high for the momentary PA measure (ω_within_ = 0.80 and ω_between_ = 0.94) and momentary NA measure (ω_within_ = 0.68 and ω_between_ = 0.95).

Participants then received the prompt: ‘Think about the interaction with other kids your age that made you feel the worst since the last beep.’ They were asked to type out details about this interaction. If participants could not think of a negative interaction, they could select an option that states, ‘I am having trouble thinking of something.’ They were then probed with follow-up questions to help them think about what happened since the last beep (e.g. ‘What were you doing when you completed the last beep?’; ‘Was there anything minor that happened that bugged you, like somebody said or did something that annoyed you, hurt your feelings just a little, or disappointed you?’). If participants continued to indicate that they did not have a negative interaction, this observation was coded as ‘no negative interaction’ and was not included in the calculation of the social threat score. Thus, the social threat score captures affective responses to experienced negative social interactions but does not inherently capture frequency of negative social interactions. Participants were then asked to indicate who was involved in the negative interaction. They could choose from the following options (with more than one selection possible): ‘friend or friends,’ ‘boyfriend/girlfriend or kid I have a crush on,’ ‘other kid(s),’ ‘sibling/stepsiblings close to my age (less than 2 years younger or older),’ ‘cousin(s),’ ‘mother/step-mother,’ ‘father/step-father,’ ‘siblings/stepsiblings NOT close to my age (greater than 2 years younger or older),’ ‘teacher or coach’ and/or ‘other adult(s).’

After typing out details about the negative interaction and identifying who was involved in the interaction, participants were asked to answer questions about how they felt during the interaction. First, they were asked to indicate how sad, worried, stressed and mad they felt during the interaction using a 0–100 sliding scale. These ratings were averaged at each observation for a measure of NA in response to the negative interaction. Internal consistencies for NA in response to the negative interactions was adequate (ω_within_ = 0.64 and ω_between_ = 0.88).

**Fig. 1.**
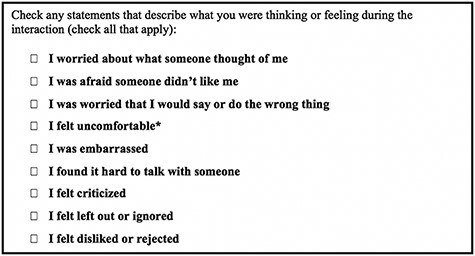
EMA social threat statements.

Note. *This item did not load significantly on a social threat score and thus was excluded from analyses.

Participants were then given a checklist that included statements that describe how they may have been thinking or feeling during the interaction (referred to as ‘social threat statements’) and were asked to check off which statements applied to them in the situation (Figure 1). Examples of social threat statements include, ‘I felt criticized’ and ‘I felt disliked or rejected.’ These questions took approximately 5 min to complete at each interval. Internal consistencies for this nine-item social threat measure were ω_within_ = 0.64 and ω_between_ = 0.91. These statements were used to create a ‘perceived social threat score’ for each participant (see ‘EMA Perceived Social Threat Data Analysis’).

#### Chatroom interact task.

The Chatroom Interact Task is an fMRI task used to examine neural responses to peer rejection and acceptance (Silk *et al.*, 2012b, 2014). The first component of the task was completed in the laboratory several weeks before the fMRI scan. During this laboratory visit, participants were shown photographs and fictitious biographical profiles of other girls their age (virtual peers) other girls whom they are told are also participating in the study. Participants also provided their own photographs and profiles to increase believability and engagement in the task. Participants then chose the top five peers with whom they would be most interested in interacting during the fMRI scan. During the fMRI scan, adolescents were matched with the two peers they ranked highest to increase the salience of the interactions and reviewed the biographical profiles for these peers prior to starting the task.

The fMRI task is made up of four blocks with 15 trials in each block, for a total run time of 15.1 min. Stimuli were presented using E-prime 1.0 software (Psychology Software Tools, Pittsburgh, PA). During the fMRI task, participants and peers took turns selecting who they would rather talk to about different topics, such as movies, music and friends. Throughout the task, pictures of participants and peers are shown two at a time. During the first block, participants complete control trials (Olino *et al.*, 2015), in which a dot appears over one of the two faces on the screen. Participants are asked to indicate which side of the screen the dot appeared on by pressing a button. Control trials were created to address concerns that peer acceptance trials are not an ideal control with respect to rejection, given the strong social evaluative component. Blocks two through four were feedback blocks. During the second block, participants choose which peer they would rather chat with. During the third and fourth blocks, the subject is chosen/not chosen by their virtual peers. In one of these blocks, the first virtual peer chooses the participant in two-thirds of the trials (‘acceptance’ trials) and rejects the participant in one-third of the trials (‘rejection’ trials), and in the other block the second peer rejects the participant in two-thirds of the trials and chooses the participant in one-third of the trials. The order of these blocks and the trials within each block are randomized across participants. Topics are presented randomly and repeated in each block. Following each selection, the photograph of the person who is chosen is highlighted and the photograph of the person who is not chosen is superimposed with a large ‘X’. Each trial is 15 s long; the topic (i.e. ‘Who would you rather talk to about [topic]?’) is presented for 3 s and the feedback (i.e. highlight of one face and ‘X’ superimposed over the other) is presented for 12 s. To maintain task engagement, participants are asked to indicate, using a button press, whether the person on the left or right was chosen when they are not the ones choosing. Analyses focused on the rejection, acceptance and control trials.

### Procedure

The study was approved by a university Institutional Review Board and consisted of three visits to the laboratory and a home protocol. At Visit 1, parents provided informed consent and youth provided informed assent. Following consent, the WASI and K-SADS-PL were administered to determine eligibility. At Visit 2, eligible participants completed the first part of the Chatroom Interact Task and were given an android smartphone to complete the EMA home protocol. Immediately following Visit 2, participants began the EMA protocol, which lasted for 16 consecutive days (10 weekdays, 3 weekends). Following the EMA collection, youth completed the fMRI scan. Before entering the scanner, participants were trained in a simulation MRI scanner to familiarize them with the tight space and the loud sounds of the scanner.

#### fMRI acquisition.

Data were acquired using a Siemens 3T Prisma magnet with a 32-channel phased array coil. Pillows were used to minimize head movement. A PC running E-Prime (www.pstnet.com) was used to control stimulus display. Stimuli were projected onto a screen at the head of the scanner bore, viewable via a mirror attached to the head coil. Participants were equipped with a response glove on their right hand to make responses during the task. All included participants were right handed.

Anatomical images covering the entire brain were acquired first using a three-dimension magnetization-prepared rapid gradient-echo T1-weighted sequence [repetition time (TR) = 2300 ms, echo time (TE) = 3.93 ms, flip angle 9°, inversion time (TI) = 900 ms, voxel size = 1 mm^3^]. Functional images were acquired using multi-band gradient echo-planar (EPI) sequences (60 slices, three-factor multiband) sensitive to BOLD Blood Oxygen Level Dependent contrast [T2*] (TR = 1500 ms, TE = 30 ms, flip angle 55°, voxel size = 2.3 × 2.3 × 2.3 mm). Field maps were acquired using gradient EPI imaging sequence for correction of field distortions in the functional images with the following parameters: TR = 590 ms, TE1 = 4.92 ms, TE2 = 7.38 ms, voxel size = 2.3 × 2.3 × 2.3 mm, flip angle 60°. Total run time for the Chatroom Interact task was 15 min and 9 s.

### EMA perceived social threat data analysis

For this analysis, we only included negative interactions experienced with a peer (i.e. friend, girlfriend or boyfriend, other kid) and did not include interactions with family members. Data were quality checked to confirm that participants were accurately reporting on negative interactions with a peer. For example, if a participant reported a negative interaction with a peer but then wrote ‘nothing’ in the description of the event, her data for this event were not included. If a participant did not endorse at least three usable negative interactions with peers over the 16 days of EMA data collection, her data were not used in the analysis. At this threshold, data from eight participants were excluded. Excluded participants did not differ from included participants in age or anxiety symptoms at baseline. On average, participants reported a usable negative interaction with a peer on 43.0% of completed EMA observations, although variability in this percentage was high (s.d. = 24.6%, range = 5.77–100%). Included participants reported an average of 18.0 (s.d. = 10.6) negative interactions with a peer over the 16-day period.

Multilevel EFAs were completed in Mplus Version 8.4 (Muthén and Muthén, 1998–2018) to evaluate the structure of the nine-item scale to assess social threat. All items (up to 9) that loaded significantly on a social threat factor were summed for each observation. Items were summed across each observation because we assumed each item to be weighted equally, without the possibility of missing data within each observation because the items were administered in a checkbox format. To provide preliminary validation of the EMA perceived social threat scale, Mplus was used to calculate within- and between-person associations between social threat sum scores (i.e. the sum of the social threat statements endorsed for each negative peer experience) and NA in response to each negative peer interaction. Within- and between-person associations between social threat sum scores and momentary NA or PA at the start of each EMA observation were also examined.

Based on results from the EFA, an average ‘perceived social threat score’ was computed for each participant by summing the social threat statements endorsed for each negative peer interaction and averaging across the total number of observations in which a negative peer interaction was endorsed (up to 54 total observations). These perceived social threat scores were used in all neuroimaging analyses. Momentary NA ratings (reported at the beginning of each observation) were also aggregated across all observations to create a trait NA measure for each participant, for use in sensitivity analyses.

### fMRI data preprocessing and analysis

All fMRI data were preprocessed according to standard protocols based on the general linear model (GLM), using a canonical hemodynamic response function, in SPM12 (Wellcome Department of Cognitive Neurology, London, UK). The preprocessing procedure includes image reconstruction and reorientation, co-registration with the high-resolution structural image, spatial realignment and normalization to a standard Montreal Neurological Institute T1 template with 2 mm voxels, and spatial smoothing using a 6 mm full-width at half-maximum Gaussian kernel. High pass filtering (128s) was applied to remove low frequency noise in the EPI signal. Head motion artifact was detected, and ArtRepair was used to make appropriate adjustments. Scans with >0.5 mm of incremental motion, >3 mm from the baseline image and/or 3 s.d. of intensity shifts were considered outliers. Outlier scans were replaced with a linear interpolation between the two nearest non-outlier scans. Subjects with more than 25% of volumes with excess movement were excluded from analyses. These more liberal movement thresholds were chosen to maximize the size of this early adolescent sample higher in anxiety symptoms than a typical community sample due to oversampling based on shy/fearful temperament. Using this threshold, data from 19 participants were excluded. For first-level analyses, we modeled Feedback Anticipation, Choice Anticipation, Peer Acceptance Feedback, Peer Rejection Feedback, Choice Feedback and Control Feedback, consistent with an event-related design, with motion parameters included as nuisance regressors. Based on a priori hypotheses, group-level region-of-interest (ROI) approaches were used for all analyses. To correct for multiple comparisons, we first estimated intrinsic smoothness of the masked functional data using AFNI’s 3dFWHM module with the spatial autocorrelation function (acf) option. These acf parameters were applied to AFNI’s 3dClustSim module. Simulation results revealed the number of voxels needed to meet a pre-determined starting voxel-wise threshold of *P *< 0.005 and cluster threshold of *P *< 0.05 within each mask. This starting threshold was motivated by challenges in balancing potential Type I and II errors in neuroimaging (e.g. Lieberman and Cunningham, 2009); however, to increase transparency, we also present results using a voxel-wise threshold of *P *< 0.001 and cluster threshold of *P *< 0.05.

#### BOLD activation analysis.

**Fig. 2.**
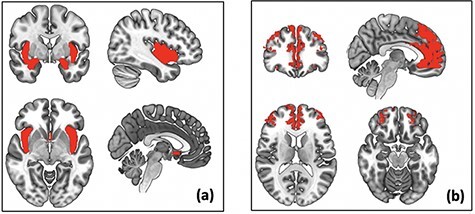
Anatomical ROI masks used in analyses (a) Social–affective ROI mask, including the anatomically defined sgACC (BA 25), bilateral insula and bilateral amygdala. (b) Gray matter mPFC ROI mask.

Anatomically defined ROI masks for the bilateral amygdala, sgACC (BA 25) and bilateral anterior insula were created using WFU PickAtlas Tool (v3.0.5b) (http://fmri.wfubmc.edu/software/pickatlas) and combined into one ‘affective-salience’ ROI mask (32240 mm^3^; Figure 2a). First-level contrasts for each participant were included in a second-level multiple regression in SPM12 to examine correlations between social threat scores and neural activity to Peer Rejection Feedback > Control Feedback within the affective–salience mask. A cluster size of 256 mm^3^ was needed for correction within this mask at a voxel-wise threshold of *P *< 0.005. Supplemental whole-brain regression analyses were also performed to explore other neural regions that may be correlated with social threat scores.

In sensitivity analyses, the total number of negative peer interactions was added as a predictor to the regression model in SPM to confirm that associations between brain function and perceived social threat reactivity held after statistically controlling for frequency of negative events, as well as to examine whether heightened neural responsivity to social rejection is also related to higher frequency of negative events in daily life. Trait NA, or the aggregate measure of NA reported at the start of every observation, was also controlled for in sensitivity analyses in SPM, to test whether neural function is associated with perceived social threat reactivity above and beyond trait levels of NA. Finally, given the novelty of the control trials, a second-level one-sample *t-*test was run in SPM to examine statistically significant activation for Peer Rejection Feedback > Control Feedback in a supplemental whole-brain analysis.

#### Functional connectivity analysis.

The CONN toolbox for SPM (Whitfield-Gabrieli and Nieto-Castanon, 2012) was used for seed-to-voxel connectivity analyses. Psychological regressors included effects of task (Feedback Anticipation, Choice Anticipation, Peer Acceptance Feedback, Peer Rejection Feedback, Choice Feedback and Control Feedback). Six head realignment motion parameters were included as nuisance regressors for each participant, and physiological noise from white matter and cerebrospinal fluid was regressed out for each participant. Linear de-trending and a 0.008–0.09 Hz temporal band-pass filter were also applied.

A first-level functional connectivity analysis (referred to as ‘weighted-GLM’ in CONN) provided weighted correlation measures of condition-specific associations between the amygdala seed BOLD timeseries and each voxel in an mPFC gray matter mask. This mask was created using the WFU PickAtlas Tool v3.0.5b and automated anatomical labeling atlas (44 176 mm^3^; Figure 2b). This mask encompassed the mPFC (ventral and dorsal) and ACC, regions implicated in emotion regulation with structural connections to the amygdala (Etkin *et al.*, 2011). A cluster size of 256 mm^3^ was needed for correction within this mask at a voxel-wise threshold of *P *< 0.005. Condition-specific weights were defined by modeling each condition of interest with a boxcar function and convolving with the canonical hemodynamic response function. This weighted GLM approach provides ‘absolute’ measures of functional connectivity occurring during a single task condition, using a nonparametric estimation of weighted correlation measures within each task condition (e.g. Belleau *et al.*, 2020). To generate amygdala–mPFC correlation maps during peer rejection feedback, the time series was extracted separately from right and left amygdala seeds, defined anatomically by the CONN toolbox and correlated with every other voxel in the ROI mask. The correlation maps were normalized using a Fischer’s *z* transformation and used in group-level statistics. A second-level regression analysis was used to examine associations between perceived social threat scores and amygdala-seeded connectivity during peer rejection feedback. This analysis was run separately for the left and right amygdala. Similar to BOLD activation analyses, sensitivity analyses were conducted controlling for the total number of peer interactions and trait NA in the CONN toolbox.

### Exploratory analyses

Secondary analyses were conducted to explore whether associations between real-world social threat reactivity (EMA) and neural reactivity were specific to neural responsivity to peer rejection feedback or whether findings might extend to neural responsivity to peer acceptance feedback and thus social evaluation generally. To do this, perceived social threat scores were regressed on neural activation to Peer Acceptance Feedback > Control Feedback and Peer Rejection Feedback > Peer Acceptance Feedback. Perceived social threat scores were also regressed on amygdala-seeded connectivity during peer acceptance feedback.

Finally, exploratory analyses examining differences in social threat scores and brain activity between temperamentally shy/fearful participants and the remainder of the sample can be found in the online supplementary materials. It should be noted that this study was not designed to examine group differences; girls high in shy/fearful temperament were overrepresented to enrich variability in threat sensitivity.

## Results

### Perceived social threat EMA measure

#### Multilevel Exploratory Factor Analyses (EFA).

Results from the EFA can be found in Table 2. Results indicate that the nine items load on a one-factor solution at both the within- and between-person level. At the within-person level, the eigenvalue for factor one was 3.78 and 1.51 for factor two. At the between-person level, the eigenvalue for factor one was 5.09 and 1.55 for factor two. The item loadings on a two-factor solution yielded results that were uninterpretable. Considering this, the strong theoretical rationale for a one-factor solution, and the significant decrease in the second eigenvalue, particularly at the between-person level, provides strong evidence for a one-factor solution. However, we did note the relatively weak loading of item T4 (‘I felt uncomfortable’) at the between-person level. As a result, we excluded item T4 from all analyses and eight items were included in the final perceived social threat score; however, we note that all of the results presented in the paper remain significant when item T4 is included in analyses.

**Table 2.** Factor loadings for the 9-item social threat scale

Item	Loading
*Within-Person*	
T1	**0.81**
T2	**0.75**
T3	**0.58**
T4	**0.33**
T5	**0.53**
T6	**0.46**
T7	**0.63**
T8	**0.45**
T9	**0.70**
*Between-Person*	
T1	**0.87**
T2	**1.00**
T3	**0.61**
T4	0.16
T5	**0.61**
T6	**0.71**
T7	**0.66**
T8	**0.72**
T9	**0.78**

*Note*: *N* = 108; Observation *N* = 1644. Loadings in bold are those considered to primarily load on that factor.

### Bivariate associations

#### Within-person associations.

Zero-order correlations between the perceived social threat sum score, NA in response to the negative peer interaction, momentary NA at the beginning of the observation and momentary PA at the beginning of the observation can be found in Table 3. In the moment, perceived social threat was modestly positively associated with NA in response to the social threat. In the moment, the perceived social threat sum score was not significantly associated with PA or NA reported at the beginning of the observation.

**Table 3.** Correlations among variables at within- and between-person levels

	Perceived Social Threat Sum Score	NA during Negative Peer Experience	Momentary NA	Momentary PA
*Within*				
Perceived Social Threat Sum Score	–			
NA during Negative Peer Experience	**0.18 [0.14, 0.23]**	–		
Momentary NA	0.04 [−.01, 0.10]	**0.28 [0.22, 0.31]**	–	
Momentary PA	−0.02 [−.06, 0.04]	0.00 [−.03, 0.05]	**−0.35 [−.39, −0.29]**	–
*Between*				
Perceived Social Threat Sum Score	–			
NA during Negative Peer Experience	**0.41 [0.21, 0.56]**	–		
Momentary NA	**0.40 [0.24, 0.57]**	**0.72 [0.59, 0.83]**	–	
Momentary PA	0.03 [−.17, 0.28]	0.06 [−.12, 0.25]	−0.08 [−.25, 0.12]	–

*Note*: *N* = 108. Observation *N* = 1644. Values in bold are those for which the credibility interval did not contain zero. Confidence intervals are presented in brackets. Perceived Social Threat Sum Score = the sum of all social threat statements endorsed following each negative peer experience.

#### Between-person associations.

As shown in Table 3, on average, perceived social threat sum scores were strongly positively associated with average momentary NA at the beginning of the observations and NA during negative peer interactions averaged across observations. There was no significant association between perceived social threat and PA, on average.

#### Descriptive statistics.

Average perceived social threat scores were normally distributed across the sample, with a range from 0.00 to 3.00, mean of 0.98 and standard deviation of 0.74, suggesting sufficient variability. NA in response to negative peer interactions was higher on average (*M *= 24.20, s.d.* *= 16.33, range = 2.69–70.06) than momentary NA measured at the time of the observation (*M *= 7.52, s.d.* *= 8.68, range = 0.04–37.59); paired samples *t*-tests also revealed that at the within-person level, participants reported higher average NA during negative peer interactions than average NA at the start of each observation [*t*(75) = −11.86, *P *< 0.001].

### Connecting neurobiology to real-world threat processing

#### BOLD activation.

**Fig. 3.**
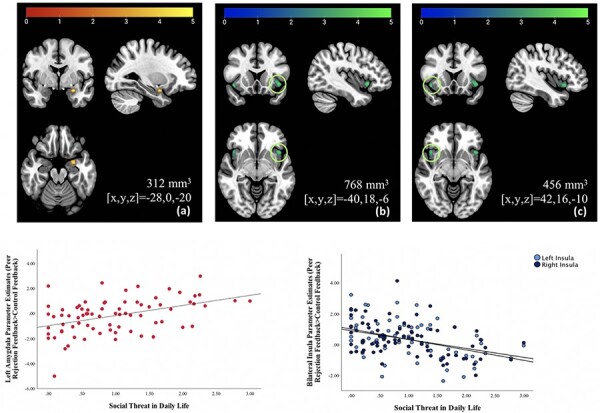
Associations between neural activation and real-world responses to perceived social threat. Perceived social threat in daily life, measured using ecological momentary assessment, correlated significantly with activation to Peer Rejection Feedback > Control Feedback in three regions within the social–affective ROI mask (displayed using radiological orientation): (a) a cluster in the left amygdala, (b) a cluster in the left insula and (c) a cluster in the right insula. Clusters positively associated with social threat at a voxel-wise threshold of *P *< 0.005 are shown with using red-yellow color scale. Clusters negatively associated with social threat at a voxel-wise threshold of *P *< 0.005 are shown with using blue-green color scale. Scatterplots are displayed for illustrative purposes.

A significant positive association between perceived social threat scores and activation in a cluster in the left amygdala (cluster size = 312 mm^3^; peak *x*,*y,z* = −28,0,−20; *Z *= 4.28) to Peer Rejection Feedback > Control Feedback was found. Significant negative associations between perceived social threat scores and activation in the left ventral anterior insula (cluster size = 768 mm^3^; peak *x*,*y,z* = −40, 18, −6 *Z *= 4.08) and right ventral anterior insula (cluster size = 456 mm^3^; peak *x*,*y,z* = 42,16,−10 *Z *= 3.89) to Peer Rejection Feedback > Control Feedback were also found (Figure 3). Associations were driven by neural activity during rejection trials, rather than by activity during control trials (Figure S1 in online supplement).

All findings surpassed a more conservative voxel-wise threshold of *P *< 0.001, for which a cluster size of 88 mm^3^ was needed to correct for multiple comparisons at a cluster threshold of *P *< 0.05 (amygdala: 168 mm^3^; left insula: 376 mm^3^; right insula: 200 mm^3^). All findings held when controlling for the total number of negative peer interactions over the 16-day period at a voxel-wise threshold of *P *< 0.001, and the total number of interactions was not significantly associated with brain activity. Controlling for trait NA at a voxel-wise threshold of *P *< 0.001, the bilateral insula findings held but the resulting amygdala cluster did not meet the cluster-wise threshold (cluster size = 72 mm^3^).

Supplemental whole-brain analysis with more liberal thresholds (i.e. voxel-wise threshold of *P *< 0.005 and cluster-level threshold of *P_FWE_ *< 0.05) noted only one additional correlation between perceived social threat scores and brain activation to Peer Rejection Feedback > Control Feedback. This negative association was found in the right parietal cortex (Brodmann area 40; Figure S2 in online supplement); no findings surpassed a whole-brain voxel-wise threshold of *P *< 0.001. Results from a one-sample *t*-test examining neural activity across the whole brain for the Peer Rejection Feedback > Control Feedback contrast can be found in the supplement (Table S1 in online supplement). This contrast elicited expected activation in regions involved in salience detection and self-referential processing, including the insula, prefrontal cortex and parietal cortex.

#### Functional connectivity.

**Fig. 4.**
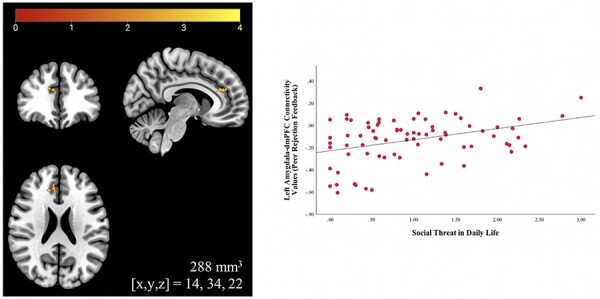
Associations between fronto-amygdala connectivity and real-world responses to perceived social threat. Perceived social threat in daily life correlated positively with functional connectivity between the left amygdala (anatomically defined) and a cluster in the right dmPFC/dorsal ACC (shown here, radiological orientation) during peer rejection feedback at a voxel-wise threshold of *P *< 0.005. Youth reporting more social threat in daily life showed less negative fronto-amygdala connectivity during rejection feedback. Scatterplot is displayed for illustrative purposes.

A significant positive association between perceived social threat scores and functional connectivity between the left amygdala and a cluster in the right dorsomedial PFC (dmPFC; Brodmann area 9) during peer rejection feedback at a voxel-wise threshold of *P *< 0.005 was found (cluster size = 288 mm^3^; peak *x*,*y,z* = 143 422; *Z *= 3.20; Figure 4). Girls reporting greater emotional reactivity to perceived social threat in daily life showed less negative left amygdala–right dmPFC connectivity to rejection. No significant associations were found with the right amygdala seed. This finding did not hold controlling for trait NA or the total number of negative peer interactions and did not survive a more conservative voxel-wise threshold of *P *< 0.001. No additional findings emerged in an exploratory whole-brain analysis.

### Exploratory analyses: extension to social acceptance

Several findings replicated for neural activation to social acceptance relative to control. At a voxel-wise threshold of *P *< 0.005, higher perceived social threat scores were associated with higher activity in the left amygdala to peer acceptance feedback relative to control feedback (cluster size = 352 mm^3^; peak *x*,*y,z* = −28,0,−20; *Z* = 4.52) and lower activity in the left insula (cluster size = 872 mm^3^; peak *x*,*y,z* = −40,18,−6; *Z* = 4.28) and right insula (cluster size = 472 mm^3^; peak *x*,*y,z* = 42,16,−10; *Z* = 3.71); all findings remained significant at a voxel-wise threshold of *P *< 0.001. Associations were driven by neural activity during acceptance trials (Figure S3 in online supplement).

Higher reactivity to perceived social threat in daily life was also related to lower activity in the inferior parietal cortex (Figure S2 in online supplement) at a voxel-wise threshold of *P *< 0.005, similar to whole-brain exploratory findings for the Peer Rejection Feedback > Control Feedback contrast. A one-sample *t*-test also revealed similar patterns of activation across the whole brain for the Peer Acceptance Feedback > Control Feedback contrast as was seen for the Peer Rejection Feedback > Control Feedback contrast (Table S2 in online supplement). No associations between perceived social threat scores and brain activity were found for the Peer Rejection Feedback > Peer Acceptance Feedback. No associations between perceived social threat and amygdala-seeded connectivity during peer acceptance feedback were found.

## Discussion

The present study uses innovative fMRI and EMA measures to link neural activity during a social interaction task to daily social experiences in adolescent females. Real-world correlates of fMRI findings were found, such that individual differences in neural responses to social evaluation were linked to differences in perceived social threat characterizing negative interactions with peers in daily life. Findings suggest that variability in neural activation on scanner-based tasks could provide meaningful insight into daily socioemotional functioning.

Addressing critical limitations of prior work, we used a realistic, salient fMRI task to investigate neural processing of interpersonal rejection and acceptance. Additionally, we developed and tested new EMA items to assess in depth how adolescents feel and think about themselves when faced with perceived social threat in daily life. As hypothesized, adolescent girls with more positive amygdala activation to peer rejection feedback (relative to control) reported more self-focused negative thoughts and feelings in response to social threat in daily life (e.g. ‘I felt embarrassed,’ ‘I felt rejected’). Findings align with prior work showing that adults with greater activation in brain regions that process social threat, including the amygdala, report greater social distress in daily life (Eisenberger *et al.*, 2007). Current findings were restricted to the left amygdala, which could represent meaningful left-lateralization of the amygdala response to negative emotions (Wager *et al.*, 2003). Detection of meaningful left but not right amygdala response could reflect differences in rates of habituation to emotional stimuli (Phillips *et al.*, 2001; Wright *et al.*, 2001); however, it could also reflect inadvertent effects of data preprocessing (Murphy *et al.*, 2020).

Interestingly, positive associations were also found between perceived social threat in daily life and amygdala activation to positive peer feedback. This aligns with the role of the amygdala in responding to emotional, salient stimuli with both positive and negative valence (Murray, 2007). Heightened emotional reactivity to social threat in daily life may thus be supported by heightened neural activity to social evaluation more generally, rather than heightened neural activity to social rejection specifically. Of note, research shows heightened amygdala activation to both negative and positive social evaluation in individuals with depression (e.g. Davey *et al.*, 2011; Kumar *et al.*, 2017). Adolescent girls with higher amygdala activation to social evaluation, both positive and negative, may find social evaluation highly salient and emotionally arousing and may thus care more about how others perceive them. High sensitivity to social evaluative could confer risk for depression, particularly for adolescent girls who experience more negative peer interactions and who are at greater risk for depression, including adolescents high in shy or inhibited temperament (Gladstone and Parker, 2006; Kingery *et al.*, 2010), who made up the majority of the present sample. As this sample is currently being followed longitudinally, future research will examine how altered neural activation to social evaluation supporting heightened emotional reactivity to real-world social threat confers risk for psychopathology throughout adolescence.

Negative correlations between perceived social threat scores and anterior–ventral insula activation to both rejection and acceptance were unexpected. The anterior insula plays a key role in salience processing and socioemotional processing (Uddin, 2015; Uddin *et al.*, 2017). Activation in the insula has previously been correlated with self-report measures of distress, leading some researchers to consider this region as part of a ‘social pain’ network (Eisenberger, 2012). However, more recent literature has challenged this view, showing that the insula responds strongly to social acceptance as well as social rejection (Dalgleish *et al.*, 2017; Perini *et al.*, 2018). This may support the role of the insula as a ‘neural sociometer’ that tracks salient social information that may influence an individual’s social inclusion status (Dalgleish *et al.*, 2017). In this way, high insula activity to any social feedback may be healthy and adaptive, helping individuals to appropriately respond to important social cues and successfully navigate their environments. Of note, most adolescents showed significant positive insula activation to social rejection and acceptance. However, adolescents with more positive insula activation to any social feedback perceived lower social threat in daily life, which again could argue for the role of the insula as a sociometer. More broadly, whole-brain findings suggest that similar brain regions are recruited when processing social acceptance and rejection.

Exploratory whole-brain analyses also identified a negative correlation between perceived social threat scores and activation in the right inferior parietal cortex to social rejection and social acceptance relative to control. The inferior parietal cortex plays a role in self-referential processing (Sajonz *et al.*, 2010), and activity in the right inferior parietal cortex specifically has been associated with self-awareness (Uddin *et al.*, 2006) and perspective-taking (Ruby and Decety, 2003), as well as processing differences in social distance (i.e. close friend *vs* acquaintance; Parkinson, Liu, and Wheatley, 2014). Thus, this region may play a role in generating awareness of how the self is connected to others (Decety and Somerville, 2003). Similar to insula findings, higher activity in this region to rejection and acceptance may be adaptive for social functioning and support healthy social relationships. Adolescents with reduced right inferior parietal cortex activation to social feedback may be more emotionally reactive to negative social evaluation because of lower interpersonal awareness, although this remains to be explored further as the parietal cortex was not a region identified a priori and did not survive a more stringent voxel-wise threshold of *P *< 0.001.

Findings from functional connectivity analyses were restricted to neural activity during social rejection, although it should be noted that this finding also did not survive a more conservative voxel-wise threshold or statistically controlling for trait NA or the total number of negative peer interactions. Girls reporting greater reactivity to perceived social threat in daily life using EMA showed less negative coupling between the amygdala and dmPFC during rejection feedback. This finding could reflect less effective prefrontal regulation over the amygdala’s response to rejection. While more ventral portions of the PFC are traditionally associated with emotion regulation (Etkin *et al.*, 2011), the dorsal PFC may work to downregulate the amygdala through more ventral regions, such as the orbitofrontal cortex (Ochsner *et al.*, 2004; Eippert *et al.*, 2007; Blair *et al.*, 2007; Frank *et al.*, 2014). Negative connectivity between the left amygdala and dmPFC has been previously implicated in conscious fear perception (Williams *et al.*, 2006). Additionally, in clinically anxious youth, altered connectivity between the amygdala and a similar dorsal PFC region during threat processing was found to correlate with EMA measures of avoidance in response to negative events (Price *et al.*, 2016). Future connectivity analyses that incorporate information about directionality (e.g. non-parametric directionality analysis; West *et al.*, 2020) may help deepen understanding of how the dorsal PFC and amygdala interact when processing social rejection.

An important contribution of this study is the testing of a novel EMA measure of social threat reactivity. This new measure allowed us to collect more detailed information on teens’ emotional reactions to perceived negative interpersonal interactions with peers. We view this perceived social threat score as a state measure that when aggregated across time indexes trait levels of sensitivity to social threat in daily life, with improved ecological validity and measurement precision. Work from the personality literature suggests that traits seem to associate with momentary manifestations of behavior such that people who have higher momentary levels on a variable also have higher trait levels (Fleeson and Gallagher, 2009; Fleeson and Jayawickreme, 2015). As expected, this measure correlated only moderately with a measure of NA at the between-person level (*r *= 0.40), suggesting that adolescent girls who are more emotionally reactive to negative social interactions also generally experience higher NA in daily life. Frequency of negative peer interactions was also notable. On average, participants reported more than one negative peer interaction per day, underscoring how common negative peer experiences are during the early adolescent period. High rates of negative peer interactions could also be related to oversampling based on shy/fearful temperament, as shyness has been linked to greater peer victimization and lower friendship quality in childhood (Kingery *et al.* 2010).

Higher social threat scores could be explained by an underlying sensitivity to all social or emotional experiences during adolescence (Nelson *et al.*, 2005; Somerville, 2013; Guyer *et al.*, 2016). However, only modest within-person associations between social threat scores and NA in response to negative peer interactions, and null within-person associations between social threat scores and momentary NA or PA, could argue against this. Nonetheless, future research could explore whether perturbed activity in similar brain regions (i.e. amygdala, insula and parietal cortex) also supports heightened emotional reactivity during positive social interactions in adolescence. As the present sample was recruited for an ongoing longitudinal study, future research using mixed-effects, longitudinal models will be used to more closely explore this social threat measure and examine how social threat scores may predict changes in social relationships or psychopathology throughout adolescence.

While the current study benefits from a large sample and more ecologically valid fMRI and EMA measures, additional limitations are important to note. First, this study included a unique sample of 11- to 13-year-old girls oversampled for shy/fearful temperament. Results may not generalize to boys, other age groups, or a more typical community sample. However, early adolescence is a key developmental period in which to study associations between social threat and brain function given the dramatic biopsychosocial changes (i.e. socio-affective brain functioning, social sensitivity) associated with puberty (Crone and Dahl, 2012). Adolescent girls are particularly sensitive to social evaluation (Rudolph and Conley, 2005) and at high risk for the development of social anxiety and depression (Merikangas *et al.*, 2010). Understanding social threat-related brain–behavior associations in this high-risk sample of early adolescents may help elucidate potential mechanisms underlying the development of internalizing disorders in females and elucidate potential targets for treatment.

Methodological limitations should also be considered. First, the Chatroom Interact task might not perfectly mimic the types of rejection that adolescent girls experience in daily life. However, its virtual platform and interactive nature reflect social media applications that are ubiquitous to adolescents. One strength of this study is the incorporation of control trials. Interestingly, correlations between social threat in daily life and brain activity were not found for the peer rejection *vs* peer acceptance contrast, which is a more typical contrast used in prior work with smaller samples (e.g. Guyer *et al.*, 2015). This could speak to prior research showing more similarities than differences in brain regions processing positive and negative social feedback (Dalgleish *et al.*, 2017; Perini *et al.*, 2018). Future research using social tasks should consider the overlap and divergence in brain regions involved in processing social threat and reward. Second, the inclusion of daily EMA minimizes recall bias, maximizes ecological validity and allows study of micro-processes that influence real-world behaviors (Shiffman *et al.*, 2007). Collecting EMA also allows for studying within-person processes; however, in the present study we were primarily interested in between-person differences in social threat reactivity, hence the creation of average scores. The use of average scores as predictors of brain activity prohibited exploration of within-person processes at play, which may be seen as a limitation of the present study. Future research with larger samples could extract neural activation from a priori regions identified in the present study for use in multilevel models exploring within-person effects. Finally, this study relies on adolescent self-report of perceived social threat experiences, and future work may benefit from using additional reporters or observational methods of social threat experiences, to allow for greater objectivity.

Overall, the present study uses ecologically relevant fMRI and EMA measures to provide evidence of real-world correlates of neural responses to social threat in adolescent females. Findings contribute to our understanding of important brain–behavior associations during a sensitive period of development. This work also points to the potential for future fMRI research to provide meaningful insight into everyday behavior and supports continued use of fMRI as an important tool in developmental research.

## Supplementary Material

nsab038_Supp
