# Supplementary material for: From scanners to cell phones: neural and real-world responses to social evaluation in adolescent girls
Source: Soc Cogn Affect Neurosci. 2021 Mar 26;16(7):657–69. doi: 10.1093/scan/nsab038 (PMC8259290; doi:10.1093/scan/nsab038)
Supplement: nsab038_Supp [file nsab038_supp.zip › suppl.docx]

Online Supplemental Information

**Supplementary Methods**

**Recruitment Information.** A total of 522 families responded to recruitment efforts and completed a brief phone or web-based screen, which included the EATQ-R (Ellis & Rothbart, 2001) to determine temperament status. One-third of participants (n=44) were recruited to be in the normal range (i.e., below 0.75 SDs above the mean) on the child- and parent-report EATQ-R shyness and fear scales; once this was achieved, participants were excluded following the web-based screen if their scores on the EATQ-R placed them in the normal range. Two-thirds of the sample (*n*=85) were recruited to be high in shy/fearful temperament. Of the 522 families screened, 235 girls ages 11-13 met preliminary inclusion criteria, which included placement in a temperament group based on the EATQ-R, being inside the 11- to 13-year-old age range, and no braces/metal in the body. Of the 235 girls who met preliminary inclusion criteria and were scheduled for an in-person clinical interview, 197 completed the clinical interview. Thirty-eight participants did not complete the first visit, either because they failed to show up (*n*=22) or cancelled (*n*=16) and were unable to be rescheduled.

**Supplemental Results**

**Neural and Behavioral Differences by Temperament Status.** As reported in the methods, two-thirds of the present sample (*n*=49) were recruited as high in shy/fearful temperament, which may confer heightened risk for future social anxiety and depression. This group was compared to the remainder of the sample with EATQ-R fear and shyness scores in the normal range (*n*=27) in perceived social threat scores and neural activity. These two groups did not differ significantly in perceived social threat scores (*t*(74)=-.44, *p*=.66). Additionally, no group differences in neural activation to Peer Rejection Feedback>Control Feedback or Peer Acceptance Feedback>Control Feedback were found in the a priori ROI mask or across the whole brain. Similarly, no group differences in amygdala-seeded functional connectivity during rejection feedback or acceptance feedback were found.

**Supplementary Tables & Figures**

Table 1S

Results from whole-brain analyses for the Peer Rejection Feedback>Control Feedback contrast (voxel-level threshold of *p*_FWE-corrected_<.05; 10-voxel extant threshold).

| Anatomical Region | k | x | y | z | BA | *t* | *p*_FWE-corr_  (cluster-level) |
| --- | --- | --- | --- | --- | --- | --- | --- |
| Left Parietal Cortex | 5144 | -46 | -48 | 46 | 40 | 11.73 | <.001 |
|  |  | -34 | -58 | 44 | 39 | 10.08 |  |
|  |  | 6 | -70 | 46 | 7 | 9.89 |  |
| Cerebellum | 3055 | -12 | -82 | -28 | -- | 10.96 | <.001 |
|  |  | -26 | -70 | -34 | -- | 9.20 |  |
|  |  | -32 | -80 | -30 | -- | 8.98 |  |
| Right Parietal Cortex | 2618 | 42 | -52 | 48 | 39 | 9.97 | <.001 |
|  |  | 36 | -66 | 48 | 39 | 8.79 |  |
|  |  | 54 | -46 | 42 | 40 | 8.73 |  |
| Left Dorsolateral Prefrontal | 1386 | -42 | 24 | 46 | 8 | 9.09 | <.001 |
| Cortex |  | -48 | 26 | 38 | 9 | 8.57 |  |
|  |  | -46 | 16 | 46 | 8 | 8.39 |  |
| Left Medial Prefrontal Cortex | 1086 | -40 | 54 | 4 | 10 | 9.05 | <.001 |
|  |  | -40 | 54 | -6 | 10 | 8.63 |  |
|  |  | -38 | 54 | 12 | 10 | 7.33 |  |
| Right Dorsolateral Prefrontal | 2496 | 40 | 10 | 42 | 8 | 8.46 | <.001 |
| Cortex |  | 0 | 34 | 48 | 8 | 8.41 |  |
|  |  | 48 | 16 | 48 | -- | 8.41 |  |
| Right Visual Association Area | 466 | 32 | -86 | 10 | 18 | 7.95 | <.001 |
|  |  | 18 | -100 | 8 | 18 | 6.38 |  |
|  |  | 30 | -88 | 22 | 19 | 6.17 |  |
| Right Medial Prefrontal Cortex | 750 | 30 | 52 | 2 | 10 | 7.66 | <.001 |
|  |  | 40 | 50 | -8 | 10 | 6.69 |  |
|  |  | 26 | 62 | 20 | -- | 6.62 |  |
| Left Visual Association Area | 354 | -18 | -98 | 10 | 18 | 7.55 | <.001 |
|  |  | -30 | -90 | 14 | 19 | 6.87 |  |
|  |  | -26 | -88 | 4 | 18 | 5.94 |  |
| Right Temporal Cortex/ | 148 | 60 | -36 | -8 | 21 | 7.47 | <.001 |
| Fusiform Gyrus |  | 52 | -48 | -10 | 37 | 5.62 |  |
| Cerebellum | 138 | 0 | -60 | -32 | -- | 7.20 | <.001 |
|  |  | 2 | -52 | -34 | -- | 6.77 |  |
|  |  | -6 | -52 | -36 | -- | 5.71 |  |
| Left Insula | 99 | -28 | 22 | -6 | 13 | 6.78 | <.001 |
| Right Dorsolateral Prefrontal | 43 | 26 | 56 | 30 | -- | 6.65 | <.001 |
| Cortex |  | 26 | 50 | 38 | 9 | 6.19 |  |
| Right Insula | 153 | 30 | 26 | -6 | 13 | 6.51 | <.001 |
|  |  | 40 | 26 | -6 | 47 | 6.38 |  |
| Left Temporal Cortex | 80 | -60 | -34 | -10 | 21 | 6.40 | <.001 |
|  |  | -60 | -42 | -6 | 21 | 5.99 |  |
| Left Fusiform Gyrus | 35 | -56 | -54 | -14 | 37 | 6.35 | <.001 |

*Note.* BA = Brodmann area; k = cluster size; x,y,z = MNI coordinates; PFC = prefrontal cortex.

Table 2S

Results from whole-brain analyses for the Peer Acceptance Feedback>Control Feedback contrast (voxel-level threshold of *p*_FWE-corrected_<.05; 10-voxel extant threshold).

| Anatomical Region | k | x | y | z | BA | *t* | *p*_FWE-corr_  (cluster-level) |
| --- | --- | --- | --- | --- | --- | --- | --- |
| Left Parietal Cortex | 5258 | -46 | -48 | 46 | 40 | 12.18 | <.001 |
|  |  | -32 | -66 | 46 | 7 | 10.23 |  |
|  |  | -32 | -58 | 42 | 39 | 9.98 |  |
| Cerebellum | 3170 | -14 | -82 | -26 | -- | 10.89 | <.001 |
|  |  | -12 | -78 | -34 | -- | 10.10 |  |
|  |  | -28 | -80 | -22 | -- | 9.12 |  |
| Right Parietal Cortex | 2731 | 42 | -54 | 50 | 39 | 10.19 | <.001 |
|  |  | 34 | -70 | 48 | 39 | 9.22 |  |
|  |  | 40 | -62 | 42 | 39 | 8.76 |  |
| Left Medial Prefrontal Cortex | 1082 | -40 | 54 | 4 | 10 | 9.51 | <.001 |
|  |  | -40 | 54 | -6 | 10 | 8.61 |  |
|  |  | -38 | 54 | 12 | 10 | 7.73 |  |
| Left Dorsolateral Prefrontal | 1416 | -48 | 14 | 46 | 8 | 8.93 | <.001 |
| Cortex |  | -42 | 26 | 44 | 8 | 8.85 |  |
|  |  | -48 | 26 | 38 | 9 | 8.46 |  |
| Right Dorsolateral Prefrontal | 2740 | -2 | 32 | 48 | 8 | 8.62 | <.001 |
| Cortex |  | 48 | 16 | 48 | -- | 8.34 |  |
|  |  | 42 | 12 | 44 | 8 | 8.20 |  |
| Left Visual Association Area | 552 | -20 | -98 | 12 | 18 | 8.24 | <.001 |
|  |  | -28 | -90 | 16 | 19 | 8.03 |  |
|  |  | -26 | -88 | 4 | 18 | 6.95 |  |
| Right Visual Association Area | 377 | 32 | -86 | 10 | 18 | 8.07 | <.001 |
|  |  | 18 | -100 | 8 | 18 | 6.19 |  |
|  |  | 30 | -88 | 22 | 19 | 6.09 |  |
| Right Medial Prefrontal Cortex | 783 | 30 | 52 | 4 | 10 | 7.86 | <.001 |
|  |  | 40 | 48 | -8 | 10 | 7.15 |  |
|  |  | 24 | 62 | 20 | -- | 6.46 |  |
| Right Temporal Cortex/ | 214 | 62 | -38 | -6 | 21 | 7.67 | <.001 |
| Fusiform Gyrus |  | 62 | -46 | -4 | 37 | 6.66 |  |
|  |  | 54 | -48 | -8 | 37 | 6.20 |  |
| Left Insula | 88 | -30 | 24 | -6 | 13 | 6.68 | <.001 |
| Left Fusiform Gyrus | 50 | -54 | -52 | -14 | 37 | 6.58 | <.001 |
| Right Dorsolateral Prefrontal | 29 | 26 | 56 | 30 | -- | 6.58 | <.001 |
| Cortex |  | 26 | 50 | 38 | 9 | 5.92 |  |
| Cerebellum | 100 | 0 | -60 | -32 | -- | 6.47 | <.001 |
|  |  | 2 | -52 | -34 | -- | 6.30 |  |
|  |  | -8 | -52 | -38 | -- | 5.35 |  |
| Left Temporal Cortex/ | 91 | -56 | -30 | -12 | 21 | 6.46 | <.001 |
| Fusiform Gyrus |  | -64 | -34 | -8 | 21 | 6.15 |  |
|  |  | -60 | -48 | -4 | 37 | 6.12 |  |
| Right Insula | 165 | 30 | 26 | -6 | 13 | 6.42 | <.001 |
|  |  | 40 | 26 | -6 | 47 | 6.32 |  |
| Left Caudate | 10 | -16 | 2 | 20 | 48 | 5.81 | .004 |
|  | 12 | 42 | 46 | 24 | 9 | 5.58 |  |

*Note.* BA = Brodmann area; k = cluster size; x,y,z = MNI coordinates; PFC = prefrontal cortex.

Figure 1S

Average parameter estimates were extracted from each of the clusters that resulted from the main analysis regressing social threat scores on the Peer Rejection Feedback>Control Feedback contrast (i.e., left amygdala, left insula, right insula) during rejection trials and control trials separately, to test the extent to which the associations between social threat scores and brain activity were explained by neural activation to peer rejection feedback or control feedback. As seen below, findings were driven almost entirely by amygdala and insula activation to peer rejection feedback.

a) Left Amygdala (312 mm^3^; peak x,y,z = -28, 0, -20)


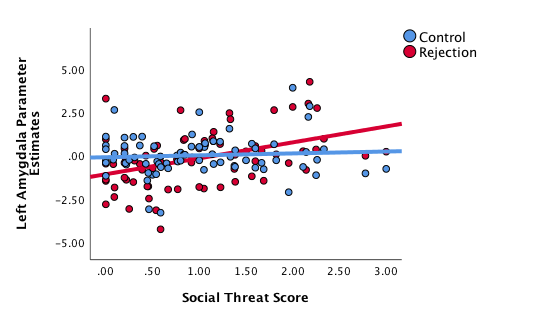


*r* = .43, *p* < .001

*r* = .07, *p* = .541

b) Left Insula (768 mm^3^; peak x,y,z = -40, 18, -6)


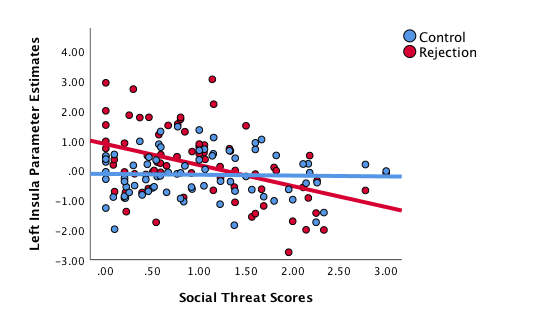


*r* = -.44, *p* < .001

*r* = -.03, *p* = .816

c) Right Insula (456 mm^3^; peak x,y,z = 42, 16, -10)


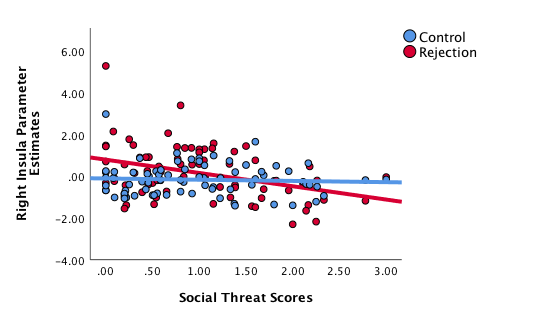


*r* = -.37, *p* = .001

*r* = -.06, *p* = .621

Figure 2S

Results from a whole-brain regression analysis with social threat EMA scores (*p*<.005 voxel-wise threshold). This analysis revealed an additional significant negative association between social threat scores and a cluster in the right parietal cortex to Peer Rejection Feedback>Control Feedback (A below: MNI coordinates (x,y,z)= 58, -28, 48; k=473; *Z*=3.93; *p*_FWE-corrected_=0.011) and Peer Acceptance Feedback>Control Feedback (B below: MNI coordinates (x,y,z)= 58, -26, 48; k=501; *Z*=3.87; *p*_FWE-corrected_=0.008).


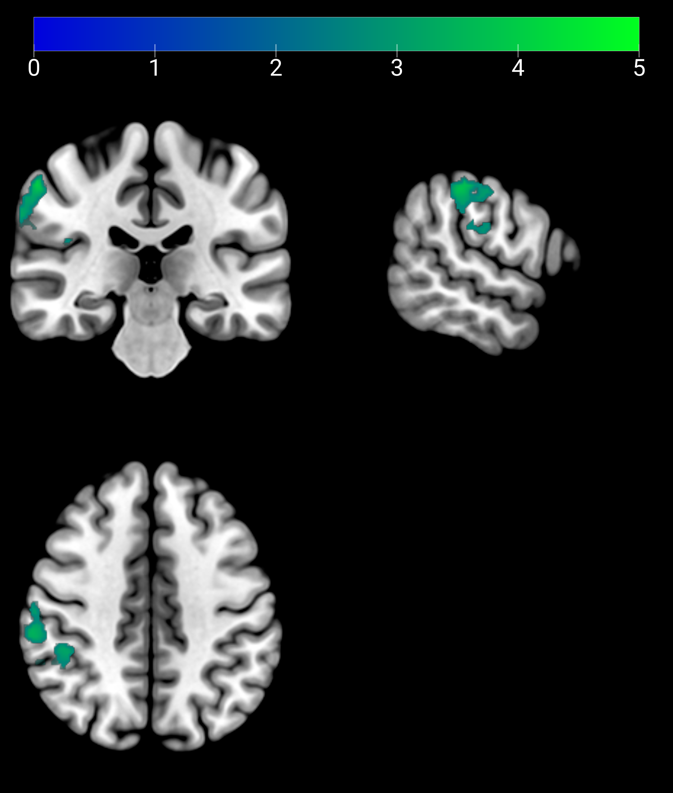

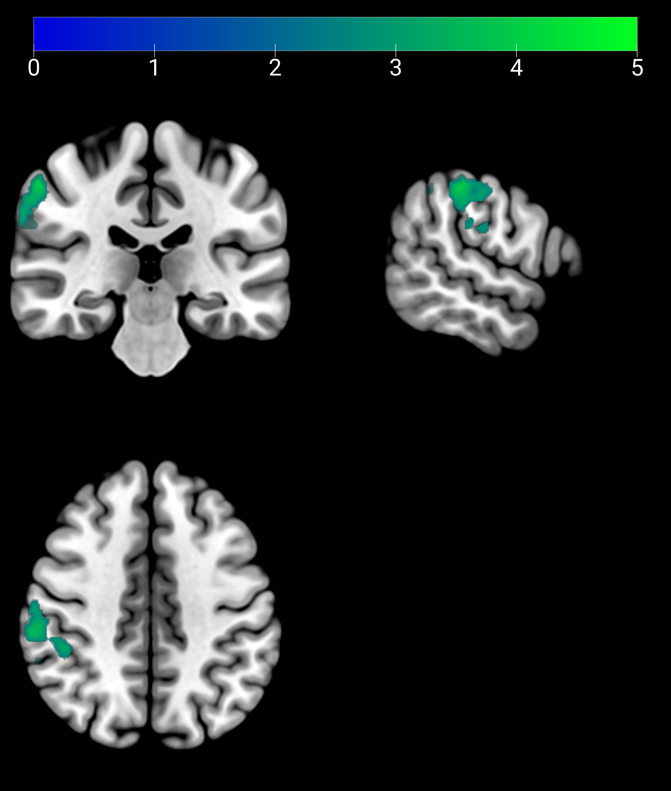


B (acceptance>control)

A (rejection>control)

Figure 3S

Average parameter estimates were extracted from the clusters that resulted from the main analysis regressing social threat scores on the Peer Acceptance Feedback>Control Feedback contrast (i.e., left amygdala, left insula, right insula) during acceptance trials and control trials separately, to test the extent to which the associations between social threat scores and brain activity were explained by neural activation to peer acceptance feedback or control feedback. As seen below, findings were driven almost entirely by amygdala and insula activation to acceptance feedback.

a) Left Amygdala (352 mm^3^; peak x,y,z = -28, 0, -20)


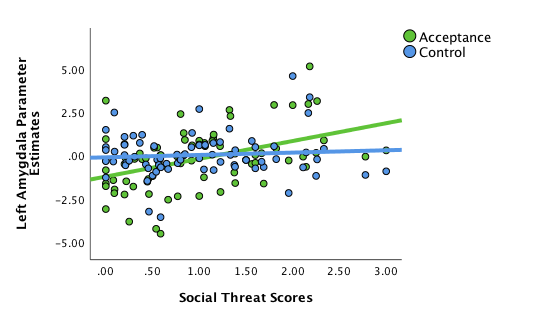


*r* = .08, *p* = .482

*r* = .43, *p* < .001

b) Left Insula (872 mm^3^; peak x,y,z = -40, 18, -6)


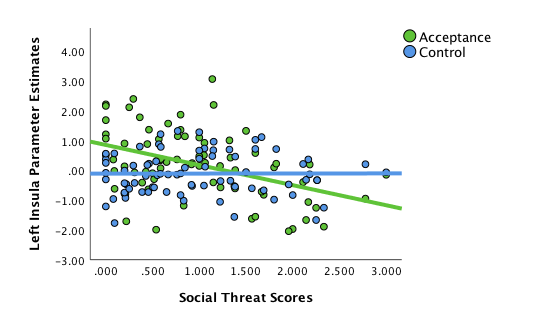


*r* = -.44, *p* < .001

*r* = .00, *p* = .982

c) Right Insula (472 mm^3^; peak x,y,z = 42, 16, -10)


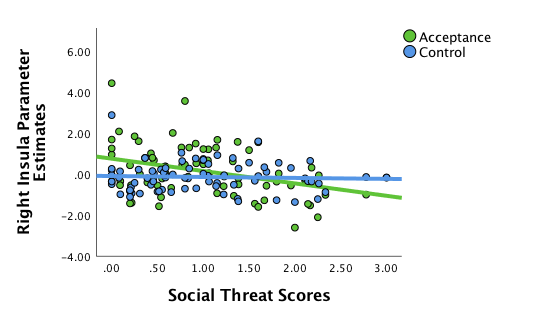


*r* = -.05, *p* = .684

*r* = -.36, *p* = .001
